# A new species of *Nebalia* (Crustacea, Leptostraca) from a hydrothermal field in Kagoshima Bay, Japan

**DOI:** 10.3897/zookeys.897.37061

**Published:** 2019-12-09

**Authors:** Takuma Hirata, Yoshihiro Fujiwara, Tomohiko Kikuchi

**Affiliations:** 1 Graduate School of Environment and Information Sciences, Yokohama National University, 79-2 Tokiwadai, Hodogaya, Yokohama, Kanagawa 240-8501, Japan Yokohama National University Yokohama Japan; 2 Japan Agency for Marine-Earth Science and Technology, 2-15 Natsushima-cho, Yokohama, Kanagawa 237-0061, Japan Japan Agency for Marine-Earth Science and Technology Yokohama Japan

**Keywords:** Hydrothermal vent, key, Leptostraca, Malacostraca, *
Nebalia
*, new species

## Abstract

A new species of Leptostraca, *Nebalia tagiri***sp. nov.** is described and illustrated. This species was sampled from 200 m depth at a hydrothermal field in Wakamiko Caldera of Kagoshima Bay, Japan. *Nebalia tagiri***sp. nov.** is different from known *Nebalia* species as follows: rostral length 2.4 times as long as width; article 4 of antennule with 3–5 robust distal spines; antennular scale approximately twice as long as wide; article 3 of antenna with eight spines and nine spine-like setae along proximal half, two thin setae and six spine-like setae on external lateral face, six spines and four simple setae on distal margin; article 1 of second maxilla longer than article 2; article 2 of mandibular palp with two thin setae; exopod of pleopod 1 with 21 spines along lateral margin; furcal rami longer than combined length of pleonite 7 and telson; rounded denticles of pleonite 6 and 7; anal-plates ‘shoulder’ not distinct. Furthermore, this specimen is the first genus *Nebalia* found in the hydrothermal vent. The distribution and ecology of this new species is also discussed and a key to all species of *Nebalia* is provided.

## Introduction

The genus *Nebalia* is a member of the Order Leptostraca, Suborder Nebaliacea, Family Nebaliidae. The family includes four other genera: *Dahlella* (Hessler, 1984), *Nebaliella* (Thiele, 1904), *Sarsinebalia* (Dahl, 1985), and *Speonebalia* (Bowman et al., 1985). *Nebalia* was established by Leach (1814) with type *Nebalia herbstii* from the British Isles. More than 37 species of this genus have been reported thus far from Africa (Barnard 1914; Kensley 1976; Olesen 1999; Bochert and Zettler 2012), Red Sea (Wägele 1983), Adriatic Sea (Dahl 1985), Britain-Celtic Sea (Dahl 1985), Greenland (Dahl 1985), Norway (Dahl 1985), Pakistan (Kazmi and Tirmizi 1989), Antarctic Sea (Dahl 1990), Falkland Islands (Dahl 1990), New Zealand (Dahl 1990), South Atlantic Ocean (Dahl 1990), Mexico (Escobar-Briones and Villalobos-Hiriart 1995; Ortiz et al. 2011), California (Martin et al. 1996; Vetter 1996; Haney and Martin 2000, 2005), Mediterranean Sea (Ledoyer 1997; Moreira et al. 2007, 2012; Koçak and Moreira 2015), New Caledonia (Ledoyer 2000), Northeast Atlantic (Haney et al. 2001; Moreira et al. 2003, 2009), Aegean Sea (Moreira et al. 2007), Hong Kong (Lee and Bamber 2011), South Korea (Song et al. 2012, 2013; Song and Min 2017), and Malaysia (Othman et al. 2016). Although our understanding of the morphology and distribution of the genus *Nebalia* has progressed, further investigation into the ecology of the genus is necessary.

In 2008, a survey was undertaken at the hydrothermal field of the Wakamiko Caldera in Kagoshima Bay, Japan using the deep-sea remotely operated vehicle (ROV) “Hyper-Dolphin” of JAMSTEC. Twenty-eight specimens of a previously undocumented species in the genus *Nebalia* were discovered. In this paper, we describe these specimens as *Nebalia tagiri* sp. nov. Taxonomic keys used to identify all currently known *Nebalia* species are also provided. Comments relating to the ecology and distribution of this species are also noted.

## Materials and methods

**Figure 1.**
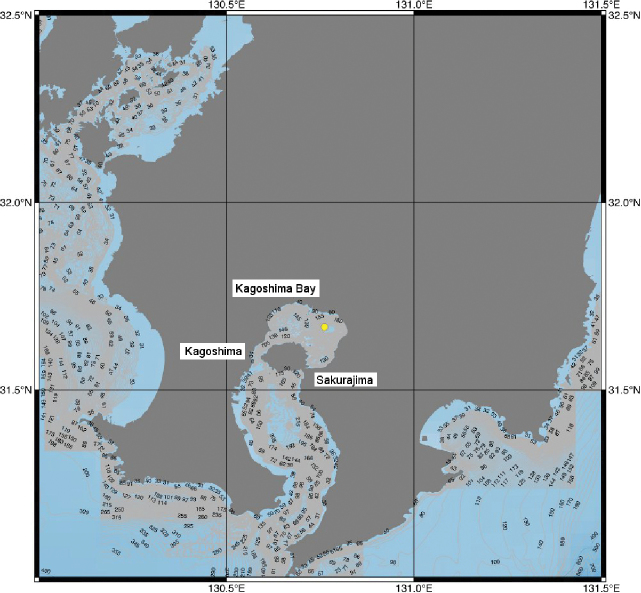
Sampling location (in yellow) of *Nebalia tagiri* sp. nov. in Kagoshima Bay.

Samples were collected in 2008 at a hydrothermal vent in the Wakamiko Caldera, Kagoshima Bay, during dive number 886 of the Japanese deep-sea ROV “Hyper-Dolphin” of JAMSTEC. The sampling site was the Wakamiko Caldera, located north of Mt. Sakurajima of Kagoshima Bay at a depth of ca. 200 m (Fig. 1). The water temperature in this field is ca. 10 °C. While bacterial mats were observed, these hydrothermal vents have no documented epifauna occurring around them. Specimens examined in this study were captured using a suction sampler installed on the ROV. Samples were sorted, identified, sexed, and measured. Total length (**TL**: measured from the articulation between the rostrum and the carapace to the posterior end of the caudal furca), carapace length (**CL**: measured from the antero-dorsal margin of carapace to the posterio-median margin of carapace), and rostrum length (**RL**: measured along the midline) were used as size indicators. Illustrations were made with the aid of a camera lucida. The type materials were deposited at the National Science Museum (Natural History), Tokyo (**NSMT**), with the remaining material deposited in the JAMSTEC Marine Biological Sample repository.

## Systematics

### Genus Nebalia

Taxon classificationAnimaliaLeptostracaNebaliidae

Leach, 1814

889A9453-B829-5E29-B219-D07900E66C51

#### Diagnosis.

Carapace almost reaching medial margin of pleonite 4. Rostrum long and narrow, 2.4 times as long as wide. Surface of eyes smooth. Article 4 of antennule with row of four simple setae and four robust distal spines. Article 3 of antenna with eight spines and nine spine-like setae along proximal half, two thin setae and six spine-like setae on external lateral face, six spines and four simple setae on terminal margin. Article 1 of endopod of second maxilla longer than article 2. Exopod of second maxilla longer than article 1 of endopod. Article 2 of mandibular palp with two thin setae. Pleonites 6 and 7 with distally rounded denticles along posterior border. Exopod of pleopod 1 with a single row of approximately 21 stout serrated spines along lateral margin. Anal plates with no distinct lateral ‘shoulder’. Furcal rami longer than combined length of pleonite 7 and telson.

### Nebalia tagiri sp. nov.

Taxon classificationAnimaliaLeptostracaNebaliidae

FE4B276F-96A7-56E3-A023-08A19615DADB

http://zoobank.org/C023E769-AF5A-4421-8671-9BFD96192723

**Figure 2.**
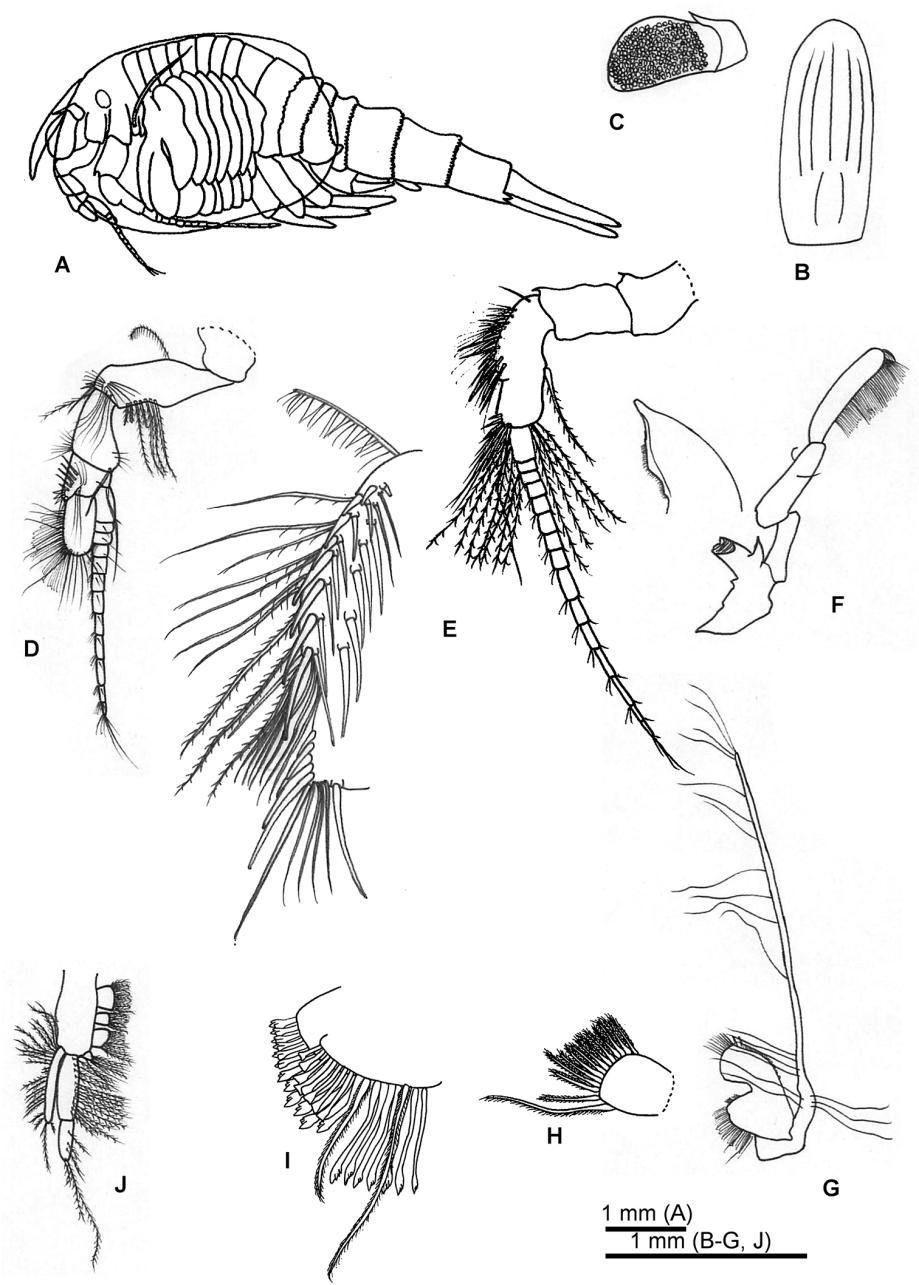
*Nebalia tagiri* sp. nov. **A** female holotype, lateral view **B** rostrum **C** eye **D** antennule **E** antenna and detail of different row of spines and setae of article 3 **F** mandible and detail of incisor process **G** first maxilla **H** detail of proximal endite of first maxilla **I** detail of distal endite of first maxilla **J** second maxilla.

**Figure 3.**
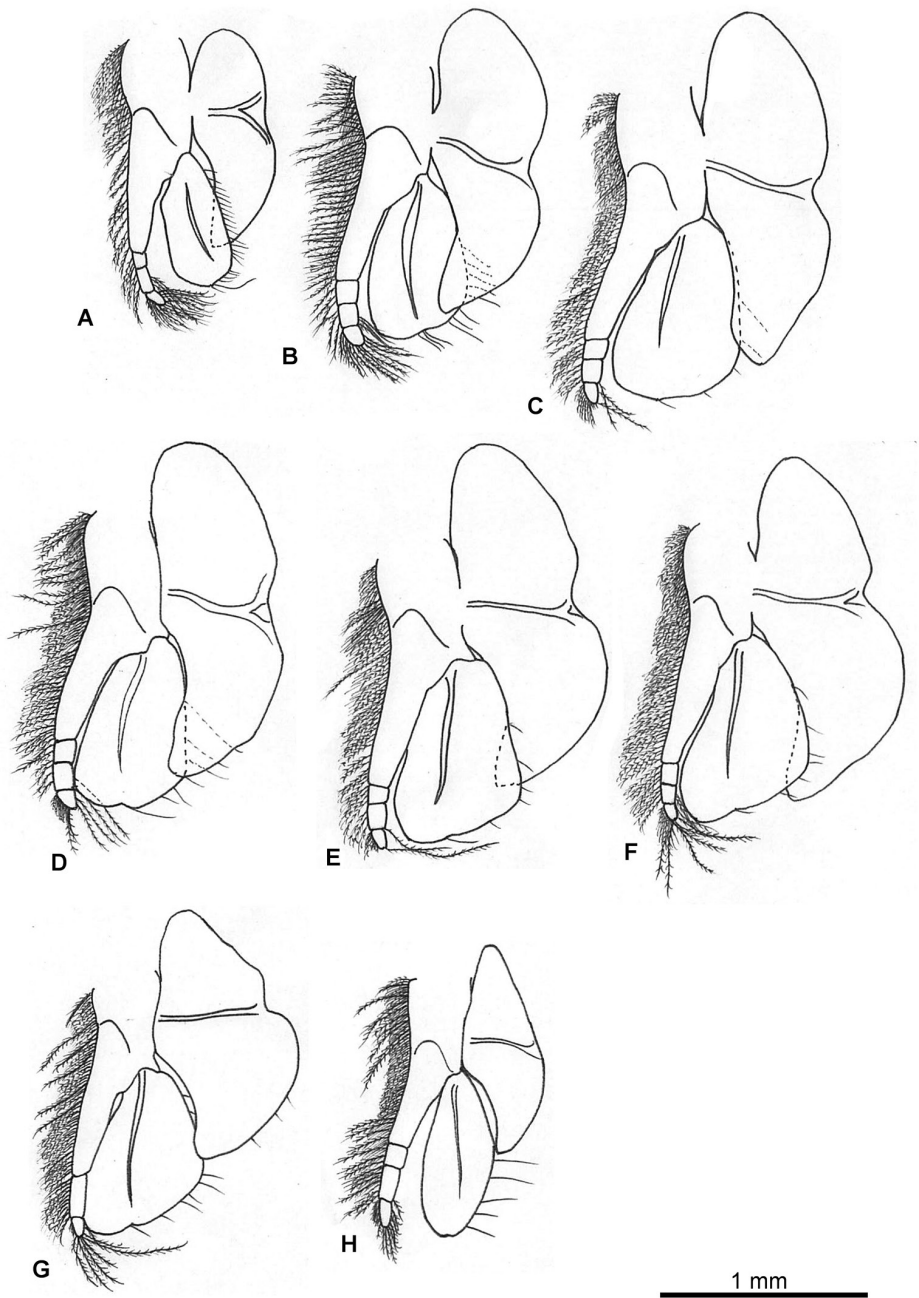
*Nebalia tagiri* sp. nov. **A** thoracopod 1 **B** thoracopod 2 **C** thoracopod 3 **D** thoracopod 4 **E** thoracopod 5 **F** thoracopod 6 **G** thoracopod 7 **H** thoracopod 8.

**Figure 4.**
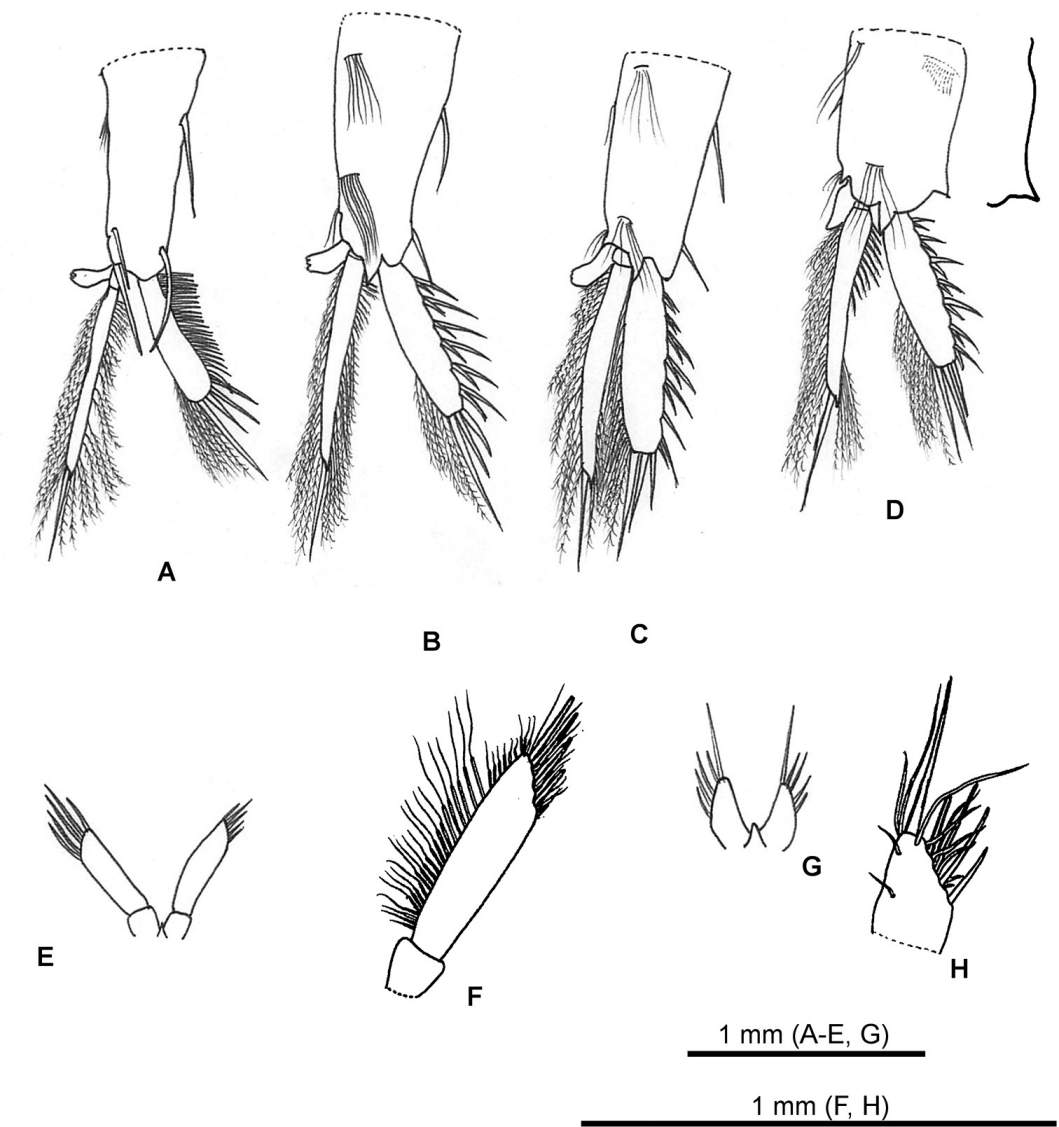
*Nebalia tagiri* sp. nov. **A** pleopod 1 **B** pleopod 2 **C** pleopod 3 **D** pleopod 4 and detail of lateral margin **E** pleopod 5 **F** detail of pleopod 5 **G** pleopod 6 **H** detail of pleopod 6.

**Figure 5.**
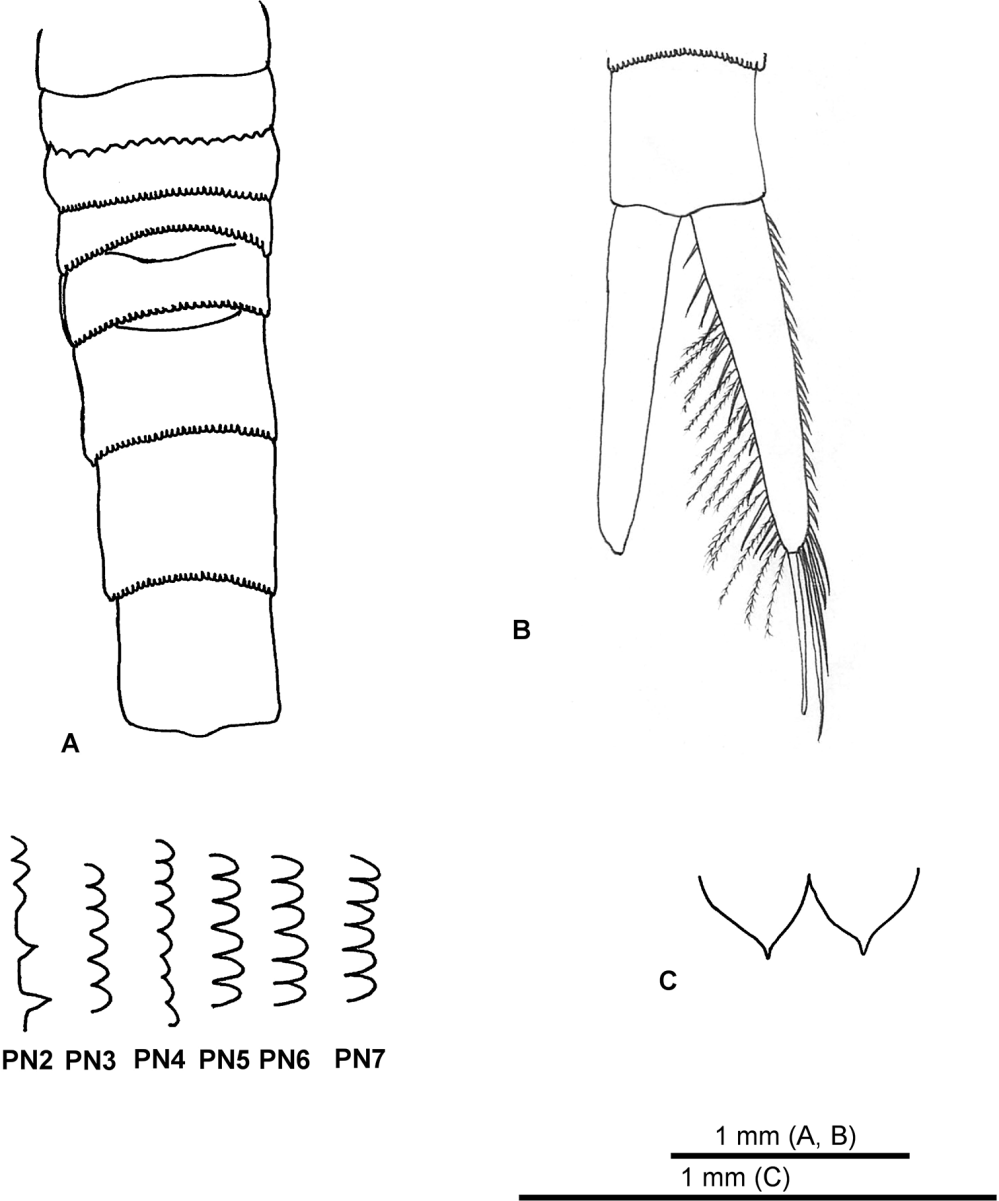
*Nebalia tagiri* sp. nov. **A** female pleonite, dorsal view and detail of denticles **B** furcal rami (setae not illustrated for left limb) **C** anal plates.

**Figure 6.**
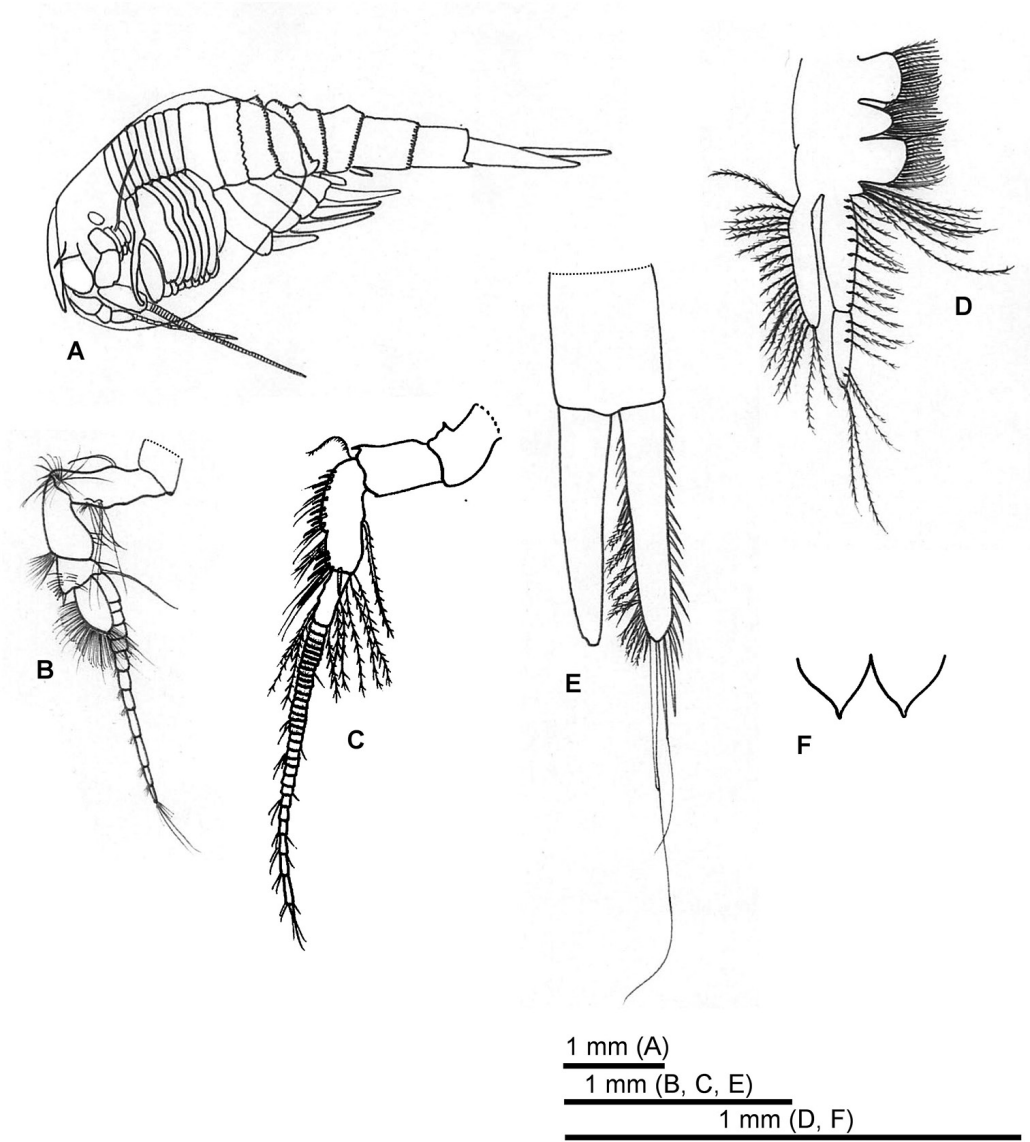
*Nebalia tagiri* sp. nov. **A** male allotype, lateral view **B** antennule **C** antenna **D** second maxilla **E** furcal rami (setae not illustrated for left limb) **F** anal plates.

**Figure 7.**
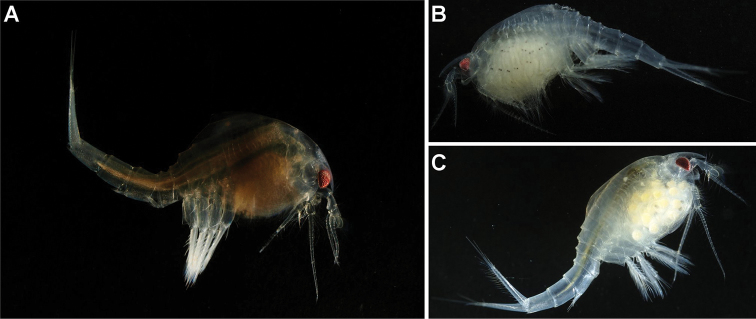
*Nebalia tagiri* sp. nov., photographs **A** female, lateral view **B** female with larvae **C** female with eggs.

Figs 2 , 3 , 4 , 5 , 6 , 7

#### Material.

Twenty-eight specimens were collected using the JAMSTEC ROV “Hyper-Dolphin” of JAMSTEC during dive 886 in the Wakamiko Caldera during the R/V “Natsushima” NT08-17 Leg-1 cruise. “Hyper-Dolphin” dive 886: the Tagiri Site on the Wakamiko Caldera: 30°40.068'N, 130°45.690'E; 200 m; 7 Aug 2008. 14 ♂♂ (TL: 3.7–6.0 mm), 13 ♀♀ (TL: 2.0–6.1 mm).

#### Types.

***Holotype***: (NSMT-Cr 26758), adult ♀ of 10.7 mm TL. ***Allotype***: (NSMT-Cr 26759), adult ♂ of 7.0 mm TL. ***Paratypes***: 3 adult ♂♂ (TL: 4.1–6.0 mm) (NSMT-Cr 26760, NSMT-Cr 26761, NSMT-Cr 26762) 3 adult ♀♀ (TL: 3.9–6.0 mm) (NSMT-Cr 26763, NSMT-Cr 26764, NSMT-Cr 26765).

#### Description.

**Female holotype. *Carapace*** (Fig. 2A) oval, ca. 1.5 times as long as wide, almost reaching pleonite 4.

***Rostrum*** (Fig. 2B) long and narrow, 2.4 times as long as width, with round apex.

***Compound eye*** (Fig. 2C): ommatidial part covering two-thirds of eye-stalk. Supraocular plate reaching to ommatidial part.

***Antennule*** (Fig. 2D): peduncle composed of four articles. Article 2 longer than article 3, with single long plumose seta on anterior margin, 5 long and three short plumose setae arising subterminally and cluster of simple setae on anterior margin, respectively. Article 3 shorter than article 2, widest distally, with terminal cluster of simple setae and long simple seta arising on anterior margin and five long plumose setae and two thin plumose setae on posterio-distal margin. Article 4 much shorter than article 3, with row of four simple setae and four robust spines distally. Antennular scale oval, twice as long as width. Flagellum slightly longer than peduncle, composed of 12 articles.

***Antenna*** (Fig. 2E): peduncle composed of 3 articles. Article 2 2.3 times as long as wide, with stout spine at dorso-distal portion. Article 3 longer than article 2, with different pattern of spines or setae along medial anterior margin as follows:

(1) proximal row of ca. six simple setae and plumose seta on inner surface;

(2) eight spines and nine spine-like setae along proximal half, the distalmost being the longest, respectively;

(3) two thin setae and six spine-like setae on external lateral face;

(4) seven thin plumose setae, three plumose setae and seven simple setae, each associated proximal spines;

(5) six robust spines increasing in length distally and four simple setae at apex, 21 long plumose setae arising from posterior distal margin, and robust plumose seta arising sub-terminally. Flagellum longer than peduncle, composed of 15 articles.

***Mandible*** (Fig. 2F) well developed. Mandibular palp composed of three articles. Article 2 equal in length as article 3, article 2 with two thin setae at mid-length on lateral margin and sub-terminal on superior margin, respectively. Article 3 cylindrical, with marginal setae-row covering four-fifths length of article. Molar process shorter than palp article 1, distal margin with row of teeth forming grinding surface. Incisor process broad basally with acute terminal process and minute tooth along lateral margin.

***First maxilla*** (Fig. 2G): proximal endite (Fig. 2H) with rounded medial margin, bearing short robust plumose setae. Distal endite (Fig. 2I) carrying two rows of stout, spatulate setae and two long plumose seta. Palp long, ca. 4.5 times longer than combined length of both endites, bearing 20 setae.

***Second maxilla*** (Fig. 2J): protopod sub-divided into four endites bearing plumose setae. Endite 1 rectangular and endite 3 rounded approximately equally sized to endite 1; endite 2 oval, smaller than endite 1 and 3; endite 4 smaller than other endites. Endopod composed of two articles, article 1 ca. twice as long as article 2, lateral margin with plumose setae, article 2 with three terminal plumose setae. Exopod reaching beyond apex of endopod article 1, bearing 23 plumose setae on lateral margin.

***Thoracopod 1*** (Fig. 3A): endopod composed of large article and three small distal articles, with numerous plumose setae along outer margin, terminal setae not reaching the terminal margin of exopod. Exopod oval, not reaching beyond the terminal article of endopod, with single long setae on terminal margin and 21 thin setae along inner margin. Epipod large, proximal lobe beyond the basis, distal lobe reaching beyond the middle of exopod.

***Thoracopod 2–6*** (Fig. 3B–F): Except for the exopod, shape of each limbs same. Eleven thin setae of inner margin of exopod in thoracopod 2, five in thoracopod 3, seven in thoracopod 4, six in thoracopod 5, seven in thoracopod 6. Shape of exopod gradually changes from oval to triangular from thoracopods 2–6.

***Thoracopod 7*** (Fig. 3G) endopod composed of one large article and two small distal articles, with numerous plumose setae along outer margin, terminal setae beyond the terminal margin of exopod. Exopod rounded and distal part expanded like a triangle, reaching beyond the terminal article of endopod, with eight thin setae along inner margin. Epipod large and triangular, proximal lobe beyond the basis, distal lobe reaching beyond the middle of exopod, with three thin setae along distal margin.

***Thoracopod 8*** (Fig. 3H): smaller than other thoracopods. Endopod composed of a large article and three small distal articles, with numerous plumose setae along outer margin, terminal setae not reaching the terminal margin of exopod. Exopod oval, reaching beyond the terminal article of endopod, with five setae along inner margin. Epipod small, proximal lobe beyond the basis, distal lobe not reaching beyond the middle of exopod.

***Pleon*** (Fig. 2A, 5A): composed of seven segments. Posterior margin of pleonite 1 smooth. Pleonite 2 with narrowly triangular denticles on half-length of posterior margin with wide flat margin. Pleonite 3 with round minute denticles. Pleonite 4 with round minute denticles, posterolateral margin expanded and forming narrow acute process. Pleonites 5, 6, and 7 with minute rounded denticles.

***Pleopod 1*** (Fig. 4A): protopod twice as long as width, with three short simple setae arising proximally, simple seta medially, two spine-like setae near base of endopod, spine-like seta near base of exopod. Endopod composed of two segments, longer than exopod, distal segment with acute process at apex, bearing long robust simple spine, lateral and medial margin each with plumose setae, nine short setae on proximal medial margin, appendix interna of proximal segment with three short recurved hooks. Exopod with row of 21 stout serrated spines along lateral margin, five stout simple spines on distolateral margin, distal one longest, plumose setae along distal inner margin.

***Pleopod 2*** (Fig. 4B): protopod 2.2 times as long as wide, six simple setae and seven simple setae on proximal and distal part of inner side, respectively; simple seta near base of exopod, with blade-like process between exopod and endopod. Endopod composed of two segments, longer than exopod, distal segment with acute process at apex, bearing long robust simple spine, lateral and medial margins each with plumose setae, ten short setae on proximal medial margin, appendix interna of proximal segment with three short recurved hooks. Exopod with row of six pairs of robust spines along lateral margin, three stout simple spines on distal margin, plumose setae along distal inner margin.

***Pleopod 3*** (Fig. 4C): protopod 2.2 times as long as wide, five simple setae on proximal and distal part of inner side respectively, simple seta near base of exopod, with blade-like process between exopod and endopod. Endopod composed of two segments, longer than exopod, distal segment with acute process at apex, bearing long robust simple spine, lateral and medial margin each with plumose setae, eight short setae on proximal medial margin, appendix interna of proximal segment with three short recurved hooks. Exopod with row of seven pairs of robust spines along lateral margin, three stout simple spines on distal margin, plumose setae along distal inner margin.

***Pleopod 4*** (Fig. 4D): protopod twice as long as width, bearing five simple setae along lateral proximal border, eleven short simple setae along ventral proximal border and four simple setae along posterior border. Posterolateral corner of peduncle produced as acute point, with blade-like process between exopod and endopod, posterior margin lacking serrations, posterolateral corner with acute projection. Endopod composed of two segments, longer than exopod, distal segment of endopod with acute process at apex, bearing long robust simple spine, lateral and medial margin each with plumose setae, ten short setae on proximal medial margin, appendix interna of proximal segment with three short recurved hooks. Exopod with row of seven pairs of robust spines along lateral margin, three stout simple spines on distal margin, plumose setae along distal inner margin.

***Pleopod 5*** (Fig. 4E, F): acute process between bases of rami. Protopod composed of two segments. Distal segment 4.5 times as long as wide, bearing five simple spines and ten short setae, lateral margin with ca. 33 simple setae.

***Pleopod 6*** (Fig. 4G, H): acute process between bases of rami, rami bearing five simple spines and six simple setae.

***Telson, anal plates, and furca*** (Fig. 5B, C): anal plates (Fig. 5C) with medial margin slightly convex, point acute, lateral margin with no distinct ‘shoulder’. Furcal rami (Fig. 5B) slightly longer than combined lengths of pleonite 7 and telson, 21 spines along outer edge, 14 robust setae and 16 plumose setae along inner margin, three long robust setae and thin spine on distolateral margin.

***Color in life*** (Fig. 7): living specimens with dark red eyes and most of body transparent.

#### Allotype

**(adult male)**: antennule flagellum more swollen proximally than in female (Fig. 6B). Antenna flagellum composed of more than 50 articles (Fig. 6C). article 2 of endopod palp half-length of article 1 (Fig. 6D). Furcal rami sub-equal in length to combined length of pleonite 7 and telson (Fig. 6E).

#### Morphological variations.

Examination of five female specimens of various sizes shows several morphological variations in the number of robust distal spines of article 4 of antennule and lateral spines of exopod of pleopod 1. Article 4 of antennule with 3–5 spines, exopod of pleopod 1 with 21–24 spines on lateral margin, respectively.

#### Etymology.

The specific name *tagiri* originates from the Japanese word ‘tagiru’ meaning “boiling”, a reflection of the hydrothermal venting and bubbling of methane and carbon dioxide as found in the habitat of *N. tagiri* sp. nov.

#### Remarks.

*Nebalia tagiri* sp. nov. differs from the other 34 described species based on four unique characteristics: (1) smooth oval eye-stalk; (2) ommatidial part covering two-thirds of eye-stalk; (3) article 4 of antennule with 3–5 robust distal spines; (4) article 1 of second maxilla endopod longer than article 2. *Nebalia tagiri* sp. nov. can easily be distinguished from *N. bipes* (Fabricius, 1780), *N. mortoni* (Lee & Bamber, 2011), and *N. koreana* (Song et al., 2012) based on the following: (1) length of rostrum in *N. tagiri*, *N. koreana*, and *N. mortoni* ca. 2.4 times as long as width, *N. bipes* approximately twice times as long as width; (2) number of distal spines of article 4 of antennule is four in *N. tagiri* sp. nov. and *N. mortoni*, three in *N. bipes* and five in *N. koreana*; (3) length of antennular scale in *N. tagiri*, *N. koreana* and *N. bipes* ca. twice as long as width, *N. mortoni* ca. 2.7 times as long as width; (4) the different pattern of spines or setae of article 3 of antenna among related three species are summarized in Table. 1.

**Table 1.** Comparison of *Nebalia tagiri* sp. nov. with related species of *Nebalia*. Key: **ro** = Rostrum; **an1** = antennule; **an2** = antenna; **mp** = mandibular palp; **pp** = pleopod; **pn** = pleonite; **a** = article; **exp** = exopod; **sp** = spine; **se** = seta; **sls** = spine-like seta; **ts** = thin seta; **ps** = plumose seta.

	**Habitat**	**Depth**	**Ro width**	**Shape of eye-stalk**	**Distal sp of an1 a4**	**Proximalrow of an2 a3**	**Lateral row of an2 a3**	**Distal row of an2 a3**	**Ts of mp2 a2**	**Sp on pp1 exp**	**Uropod length**	**Shape of pn6–7 denticles**	**Presence of ‘shoulder’ on anal–plates**	**Reference**
*N. tagiri* sp. nov.	Hydrothermal vent chimney	200 m	2.4 times	Oval	4 sp	8 sp	2 ts	6 sp	2 ts	21–24 sp	> Pn7+t	Round	No distinct	This paper
						9 sls	6 sls	4 se						
*N. abyssicola*	Mud	680–820 m	–	Oval	1 sp	8 sp	2 ts	5 sp	2 ts	25–30 sp	> Pn7+t	Round	None	Ledoyer (1997)
						6 se	6 sp	6 se						
							3 sls							
*N. bipes*	Clay and stones	5–13 m	2 times	Oval	3 sp	–	–	–	1 ts	30 sp	= Pn7+t	Round	Distinct	Dahl (1985)
*N. borealis*	Sand	240 m	2.1 times	Oval	2 sp	–	–	–	1 ts	24 sp	≥ Pn7+t	Round to acute	Distinct	Dahl (1985)
*N. koreana*	Algal mat	6 m	2.4 times	Oval	5 sp	10 sp	2 ts	5 sp	2 ts	30–38 sp	< Pn7+t	Round	No distinct	Song et al. (2012)
						6 sls	6 sls	4 se						
*N. mortoni*	–	17 m	2.4 times	Oval	4 sp	7 sp	2 ts	6 sp	2 ts	25 sp	= Pn7+t	Square	No distinct	Lee and Bamber (2011)
							4 sls							
*N. schizophthalma*	–	2886 m	2.6 times	Bilobed	5 ts	10 sls	10 se	6 sp	2 ts	15 sp	< Pn7+t	Acute	None	Haney et al. (2001)
								8 se						

*Nebalia tagiri* sp. nov. showed different characteristics from *N. koreana* or *N. mortoni* in the following points: (1) number of spines or spine-like setae along proximal half in comparison with *N. koreana* and *N. mortoni*; (2) existence of spine-like setae on external lateral face in comparison with *N. mortoni*; (3) number of spines and setae on terminal margin in comparison with *N. koreana* and *N. mortoni*, *N. bipes* is lacking detailed description; (5) thin seta of article 2 of mandibular palp is two in *N. tagiri* sp. nov., *N. koreana* and *N. mortoni* and one in *N. bipes*; (6) number of lateral spines of exopod of pleopod 1 is 21–24 in *N. tagiri* sp. nov, 25 in *N. mortoni*, and more than 30 in *N. bipes* and *N. koreana*; (7) furcal rami are longer than the combined length of pleonite 7 and the telson in *N. tagiri*. sp. nov. and of the same length in *N. bipes* and *N. mortoni*, while in *N. koreana* they are shorter; (8) denticles of posterior margin of pleonites 6 and 7 are rounded in *N. tagiri* sp. nov., *N. bipes*, and *N. koreana* while in *N. mortoni* are square-shaped.

Most species of *Nebalia* have been reported from shallow water (< 10 m depth) in the world oceans with two exceptions, *i.e.*, *Nebalia abyssicola* (Ledoyer, 1997) and *N. schizophthlma* (Haney et al., 2001) have been reported from ca. 100 m or deeper (Table 1). *Nebalia schizophthalma* was reported at a depth of 2886 m in the North Atlantic Ocean, which is the deepest record of this genus. *Nebalia tagiri* sp. nov. was collected from a hydrothermal vent chimney at a depth of 200 m in Wakamiko Caldera of Kagoshima Bay, Japan (Fig. 1). This depth of 200 m is relatively deep compared to similar species of this genus. The genus *Nebalia* has not been previously reported from any hydrothermal fields to date. The only species reported from a deep-sea hydrothermal field is *Dahlella caldariensis* (Hessler, 1984), observed on the mussel beds of hydrothermal vent areas at the Galapagos and the East Pacific Rise at depths deeper than 2000 m. On the other hand, *N. tagiri* sp. nov. was collected near the interior of chimney walls, which were close to the chimney vents spouting thermal water (ca. 200 °C).

This species was clearly observed on the inner surface of the chimney, suggesting the species is adapted to the hydrothermal environment. For a more accurate understanding of this species, further investigations are needed to determine their ecological and/or physiological aspects in relation to the hydrothermal fields.

##### Key to species of genus *Nebalia*

**Table:** 

1	Pleopod 6 composed of two segments	***N. biarticulata* Ledoyer, 1997**
–	Pleopod 6 composed of one segment	**2**
2	Entire surface of eye smooth	**13**
–	Eye surface different	**3**
3	Dorsal margin of eye smooth, with lobes only on antero-distal margin	**10**
–	Eye papillae present on dorsal margin	**4**
4	Eye with several lobes on antero-distal margin	***N. cambodiana* Song et al., 2013**
–	Eye with antero-distal margin smooth, lacking lobes	**5**
5	Ommatidial part covering most of the eye-stalk	**6**
–	Ommatidial part not covering most of the eye-stalk	**7**
6	Protopod of pleopod 4 with serrations along posterior margin	***N. cannoni* Dahl, 1990**
–	Protopod of pleopod 4 lacking serrations along posterior margin	***N. longicornis* Thomson, 1879**
7	Ommatidial part covering four-fifths of eye-stalk	***N. falklandensis* Dahl, 1990**
–	Ommatidial part not covering four-fifths of eye-stalk	**8**
8	Ommatidial part covering half of eye-stalk	***N. capensis* Barnard, 1914**
–	Ommatidial part covering two-thirds of eye-stalk	**9**
9	Denticles of pleonites 6 and 7 distally acute	***N. antarctica* Dahl, 1990**
–	Denticles of pleonites 6 and 7 rounded	***N. patagonica* Dahl, 1990**
10	Anterior margin of eye-stalk with 2 lobes	**11**
–	Anterior margin of eye-stalk with 3 lobes	**12**
11	Eye-stalk with flat anterior margin between lobes	***N. daytoni* Vetter, 1996**
–	Eye-stalk no flat margin between lobes	***N. schizophthalma* Haney et al., 2001**
12	Denticles of pleonites 6 and 7 acute distally	***N. troncosoi* Moreira et al., 2003**
–	Denticles of pleonites 6 and 7 rounded	***N. pseudotroncosoi* Song et al., 2013**
13	Shape of the eye-stalk sub-rectangular	***N. abyssicola* Ledoyer, 1997**
–	Shape of the eye-stalk oval	**14**
14	Ommatidial part covering half of the eye-stalk	**15**
–	Ommatidial part not covering half of the eye-stalk	**20**
15	Supraocular plate covering proximal portion of eye-stalk	**16**
–	Supraocular plate covering the half of eye-stalk	***N. deborahae* Bochert & Zettler, 2012**
16	Exopod of second maxilla clearly extend beyond the endopod of article 1	***N. clausi* Dahl, 1985**
–	Exopod of second maxilla subequal length to endopod of article 1	**17**
17	Furcal rami almost the same length as combined length of telson and pleonites 6 and 7	***N. marerubri* Wägele, 1983**
–	Furcal rami shorter than combined length of telson and pleonites 6 and 7	**18**
18	Furcal rami almost the same length as combined length of telson and pleonite 7	***N. gerkenae* Haney & Martin, 2000**
–	Furcal rami longer than combined length of telson and pleonite 7	**19**
19	Article 4 of antennule with single distal spine	***N. brucei* Olesen, 1999**
–	Article 4 of antennule with one or two distal spines	***N. dahli* Kazmi & Tirmizi, 1989**
20	Ommatidial part covering more than two-thirds of eye-stalk	**21**
–	Ommatidial part covering more than three quarters of eye-stalk	**32**
21	Article 1 of endopod of second maxilla shorter than article 2	**22**
–	Article 1 of endopod of second maxilla not shorter than article 2	**23**
22	Rostrum length ca. 2.4 times as long as width	***N. lagartensis* Escobar-Briones & Villalobos-Hiriart, 1995**
–	Rostrum length ca. 1.9 times as long as width	***N. terazakii* Othman et al., 2016**
23	Article 1 of endopod of second maxilla subequal in length to article 2	***N. kocatasi* Moreira et al., 2007**
–	Article 1 of endopod of second maxilla longer than article 2	**24**
24	Denticles of pleonites 6 and 7 square	***N. mortoni* Lee & Bamber, 2011**
–	Denticles of pleonites 6 and 7 not square	**25**
25	Denticles of pleonites 6 and 7 acute	**26**
–	Denticles of pleonites 6 and 7 rounded	**27**
26	Exopod of pleopod 1 bearing 15 or 16 spines on lateral margin	***N. melanophthalma* Ledoyer, 2000**
–	Exopod of pleopod 1 bearing 26 spines on lateral margin	***N. strausi* Risso, 1826**
27	Anal plates with distinct ‘shoulder’	***N. bipes* Dahl, 1985**
–	Anal plates with no distinct ‘shoulder’	**28**
28	Protopod of pleopod 4 with serrations along posterior margin	**29**
–	Protopod of pleopod 4 lacking serration along posterior margin	**30**
29	Article 3 of antennal peduncle bearing plumose setae on external lateral face	***N. mediterranea* Kocak & Moreira, 2015**
–	Article 3 of antennal peduncle lacking plumose setae on external lateral face	***N. kensleyi* Haney & Martin, 2005**
30	Furcal rami shorter than combined length of telson and pleonite 7	***N. koreana* Song et al., 2012**
–	Furcal rami not shorter than combined length of telson and pleonite 7	**31**
31	Denticle of pleonite 2 with wide flat margin between denticles	***N. tagiri* sp. nov., This paper**
–	Denticle of pleonite 2 lacking flat margin between denticles	***N. reboredae* Moreira et al., 2009**
32	Ommatidial part covering four-fifths of eye-stalk	***N. villalobosi* Ortiz et al., 2011**
–	Ommatidial part covering three-quarters of eye-stalk	**33**
33	Denticles of pleonites 6 and 7 square-shaped	***N. ilheoensis* Kensleyi, 1976**
–	Denticles of pleonites 6 and 7 not squared	**34**
34	Denticles of pleonite 6 rounded and of pleonite 7 acute	***N. borealis* Dahl, 1985**
–	Denticles of pleonites 6 and 7 similar in shape	**35**
35	Denticles of pleonites 6 and 7 distally acute	**36**
–	Denticles of pleonites 6 and 7 rounded	**37**
36	Total length of mature female more than 10 mm, Exopod of pleopod 1 bearing more than 30 spines on lateral margin	***N. hessleri* Martin et al., 1996**
–	Total length of mature female less than 5 mm, Exopod of pleopod 1 bearing 15 or 16 spines on lateral margin	***N. neocaledoniensis* Ledoyer, 2000**
37	Article 2 of mandibular palp with single thin seta, anal plate with no distinct “shoulder”	***N. herbstii* Leach, 1814**
–	Article 2 of mandibular palp with two thin setae, anal plate with distinct “shoulder”	***N. dolsandoensis* Song & Min, 2016**

## Supplementary Material

XML Treatment for Nebalia

XML Treatment for Nebalia tagiri
